# Peptide YY Regulates Bone Remodeling in Mice: A Link between Gut and Skeletal Biology

**DOI:** 10.1371/journal.pone.0040038

**Published:** 2012-07-06

**Authors:** Iris P. L. Wong, Frank Driessler, Ee Cheng Khor, Yan-Chuan Shi, Birgit Hörmer, Amy D. Nguyen, Ronaldo F. Enriquez, John A. Eisman, Amanda Sainsbury, Herbert Herzog, Paul A. Baldock

**Affiliations:** 1 Neuroscience Program, Garvan Institute of Medical Research, Sydney, New South Wales, Australia; 2 Osteoporosis and Bone Biology, Garvan Institute of Medical Research, Sydney, New South Wales, Australia; 3 Faculty of Medicine, University of New South Wales, Sydney, Australia; 4 School of Medical Sciences, University of New South Wales, Sydney, Australia; Inserm U606 and University Paris Diderot, France

## Abstract

**Background & Aims:**

Gastrointestinal peptides are increasingly being linked to processes controlling the maintenance of bone mass. Peptide YY (PYY), a gut-derived satiety peptide of the neuropeptide Y family, is upregulated in some states that also display low bone mass. Importantly, PYY has high affinity for Y-receptors, particularly Y1R and Y2R, which are known to regulate bone mass. Anorexic conditions and bariatric surgery for obesity influence circulating levels of PYY and have a negative impact on bone mass, but the precise mechanism behind this is unclear. We thus examined whether alterations in PYY expression affect bone mass.

**Methods:**

Bone microstructure and cellular activity were analyzed in germline PYY knockout and conditional adult-onset PYY over-expressing mice at lumbar and femoral sites using histomorphometry and micro-computed tomography.

**Results:**

PYY displayed a negative relationship with osteoblast activity. Male and female PYY knockout mice showed enhanced osteoblast activity, with greater cancellous bone mass. Conversely, PYY over-expression lowered osteoblast activity *in vivo*, via a direct Y1 receptor mediated mechanism involving MAPK stimulation evident *in vitro*. In contrast to PYY knockout mice, PYY over expression also altered bone resorption, as indicated by greater osteoclast surface, despite the lack of Y-receptor expression in osteoclastic cells. While evident in both sexes, cellular changes were generally more pronounced in females.

**Conclusions:**

These data demonstrate that the gut peptide PYY is critical for the control of bone remodeling. This regulatory axis from the intestine to bone has the potential to contribute to the marked bone loss observed in situations of extreme weight loss and higher circulating PYY levels, such as anorexia and bariatric obesity surgery, and may be important in the maintenance of bone mass in the general population.

## Introduction

Osteoporosis is a serious health condition presenting a major economic burden on health care systems worldwide. It is estimated that osteoporotic fracture will occur in one in two women and one in three men over the age of 60 [1]. While the etiology of osteoporosis has been viewed primarily in light of endocrine factors originating from the hypothalamo-pituitary axis, in recent years a novel endocrine axis has emerged involving peptides released from the gut [2]. Indeed, in addition to its roles in regulating energy homeostasis and appetite [3], [4], glucose metabolism [5], the neural system [6] and factors associated with higher functions such as mood and depression [7], the gut is emerging as a fundamental regulator of bone health. It is becoming increasingly apparent that a more detailed appreciation of the gastrointestinal/bone interface is vital for an up-to-date understanding of skeletal regulation, and the endocrine actions of the gut.

In light of the widespread health issues associated with osteoporosis, it is concerning that significant loss of bone is also evident in younger individuals in association with altered energy homeostasis. In particular, the low bone mass of anorexia nervosa, and the marked bone loss observed post-bariatric surgery illustrate the powerful relationship between food intake and bone. While it is known that weight reduction associated with anorexia nervosa and bariatric surgery leads to bone loss [8], [9], it is increasingly recognized that additional pathways contribute to the loss of bone resulting from altered nutritional status. Indeed, studies post bariatric surgery have reported that weight accounted for as little as 14% of the loss of bone mineral content, indicating a significant contribution by non-weight bearing factors [10].

Recent literature from human studies suggests that a ligand of the neuropeptide Y family, the gut hormone peptide YY (PYY), may play an important role in regulation of bone mass, particularly under conditions of altered energy balance [11], [12], [13], [14]. PYY, predominantly expressed in L cells in the mucosa of the ileum and colon [15], is an important component of the energy homeostatic system. Released post-prandially into the bloodstream in proportion to calorie intake, PYY acts to inhibit food intake and increase satiety [16]. Importantly, altered PYY levels are associated with several metabolic disorders which also alter bone mass, indicating a possible role for PYY in bone homeostasis. For instance, the greater circulating PYY levels in patients with anorexia nervosa are significantly associated with altered bone homeostasis and diminished bone mass [11], particularly in the spine [14]. Elevated PYY levels are a negative predictor of the bone formation serum marker, pro-collagen type I N-terminal pro-peptide (PINP) and lumbar spine bone mineral density in amenorrheic athletes [12]. Conversely, obesity is associated with lower levels of PYY [17] and greater bone mineral density [18]. However, one of the biggest changes in PYY levels is observed in patients following some forms of bariatric surgery, such as Roux-en-Y gastric bypass and gastric sleeve [19], [20], with higher post-prandial PYY levels compared to pre-surgery. Significant bone loss is also observed in obese patients following bariatric surgery [21]. However, following gastric banding, which does not alter PYY levels [22], bone loss is less pronounced [23], [24], indicating that PYY may represent a component of the non-weight related mechanisms reducing bone mass in these patients. Importantly, PYY may play a broader role in the maintenance of bone mass. A recent cross-sectional study in healthy premenopausal women demonstrated that PYY levels have a significant, negative association with total body and hip bone mass [13], explaining nearly 9% of the variance in hip bone mineral density in these women. Thus it is clear that a greater understanding of the relationship between PYY and bone is required, particularly with regard to the skeletal effect of elevated PYY levels.

Although evidence of a negative correlation between PYY levels and bone mass is emerging, this relationship is at present based on associations and is confounded by factors such as body mass index [18] and gonadal status [25]. Therefore in this study, the role of PYY on bone homeostasis was examined in mouse models with either germline PYY knockout or adult-onset conditional PYY over-expression, to assess the impact of the absence or excess of PYY, respectively, on the skeleton.

## Materials and Methods

### Animals

All research and animal care procedures were approved by the Garvan Institute/St Vincent’s Hospital Animal Experimentation Ethics Committee. Mice were housed at 22°C with a 12 h light/dark cycle. Mice had *ad libitum* access to water and standard chow (8% calories from fat, 21% calories from protein, 71% calories from carbohydrate, 2.6 kcal/g; Gordon’s Speciality Stock Feeds, Yanderra, Australia). Generation of PYY knockout mice was described previously [26]. Briefly, a 10.5 kb SpeI fragment containing 6 kb 5′-flanking sequence, the entire PYY gene and a 3 kb 3′-flanking sequence was used for the construction of a PYY-Cre knock-in construct. The linearized clone was transfected into ES cells derived from 129 Sv/J mice. Two positive clones were injected into C57BL/6 blastocysts, chimeric mice were bred to generate homozygous PYY-Cre knock-in mice.

### PYY Transgene Construct and Generation of Adult-onset PYY Over-expression Model (PYYtg)

A tamoxifen-inducible, *CMV*-driven Cre transgenic mouse model (Rosa-CreER^T2^) was obtained from the Jackson Laboratory (Bar Harbor, ME) [27]. Heterozygous Rosa-CreER^T2^ transgenic mice were crossed with homozygous PYY transgenic mice (PYY^lox/lox^) to generate PYY over-expressing (here called PYYtg) and non-PYY over-expressing littermate controls (here called WT). The PYY^lox/lox^ mice have the PYY gene inserted into the ubiquitously expressed *Rosa26* locus with a *CMV* promoter and a transcriptional stop cassette flanked by loxP sites. Therefore, in mice with the genotype of PYY^lox/CreERT2^ (PYYtg), tamoxifen treatment activates Cre-recombinase, which leads to the removal of the lox flanked stop cassette in front of the PYY gene bringing the *CMV* promoter in close proximity to the *PYY* gene and induce the over-expression of PYY. Littermate controls that carried only the PYY^lox/lox^ locus but no RosaCre were used as controls (WT). Tamoxifen (40 µg/g of body weight) was i.p. injected twice at 10 weeks of age, 3 days apart, and tissues were collected 6 weeks later.

Genotyping used genomic DNA isolated from tail tissue, confirmed later with liver DNA. PCR amplification of the fragment encompassing the stop cassette of the PYY transgene construct was performed using LacZ-F/Neo-RZ primers. Primer sequence, PCR conditions and expected product size for each primer pair are summarized in Table S1. Deletion of the stop cassette of the PYY transgene from tamoxifen-injected mice was confirmed with the mPYYtg-CMV/mPYY-W primers, producing a 150 bp product if the loxP flanked stop cassette has been deleted.

### RNA Extraction, Reverse Transcription-PCR (RT-PCR) and Quantitative Real-time PCR (qPCR)

Total RNA from liver and brain (hypothalamic and extra-hypothalamic tissue) of wildtype and PYYtg mice were isolated using Trizol® Reagent (Sigma) following the manufacturer’s protocol. Total RNA was treated with DNase I (Ambion, Austin, TX, USA) before reverse transcription using Superscript III First-Strand Synthesis System (Invitrogen, Mount, Waverley, VIC, Australia), following the manufacturer’s protocol.

Expression of PYY mRNA was first determined using RT-PCR with GAPDH as control (Table S1). PYY mRNA expression was further examined by qPCR (LightCycler® 480, Roche Applied Science, Germany) using SensiMix™ Probe (Bioline Australia Pty Ltd, Alexandria, NSW, Australia) with gene-specific primers. To control for variability in amplification due to differences in starting mRNA concentrations, ribosomal protein L19 (RPL-19) was used as an internal standard. The relative expression of PYY mRNA was computed using the comparative C_t_ method for relative quantification (Sequence Detection Systems Chemistry Guide, Applied Biosystems).

### Immunohistochemical Analysis of the Pancreas

PYY IHC was performed on two random 5 µm paraformaldehyde-fixed, paraffin-embedded pancreatic sections, as described previously [26]. Briefly, whole pancreas, fixed in 4% PBS-buffered paraformaldehyde overnight at 4°C before being processed and embedded in paraffin. Slides were incubated in 0.3% H_2_O_2_ in ethanol for 35 min, rinsed in PBS and blocked with 10% normal horse serum in PBS for 20 min at room temperature. Excess blocking solution was removed before incubating with our in-house monoclonal mouse PYY antiserum (diluted 1∶1000) overnight at 4°C. Slides were rinsed in PBS before incubation with goat anti-mouse IgG-biotin conjugated antibody (Sigma) (diluted 1∶1000) for 30 min at room temperature. Sections were rinsed in PBS and incubated for 30 min with ExtrAvidin®-Peroxidase (Sigma) (diluted 1∶250) at room temperature. After washing with PBS, immunoperoxidase staining was performed by treating the sections with diaminobenzidine (DAKO Corp., Carpinteria, California, USA) for 3 min. Slides were counterstained with haematoxylin, then dehydrated through graded ethanols and xylene before coverslipping.

### Bone Densitometry

Whole body bone mineral density (BMD) and bone mineral content (BMC) were measured with mice ventral side down (head and tail inclusion), using a dedicated mouse dual energy X-ray absorptiometer (DXA) (Lunar Piximus II, GE Medical Systems, Madison WI), as previously described [28].

### Tissue Collection and Bone Histomorphometry

For tissue collection, germline PYY knockout mice were culled at 14 weeks, PYY transgenic mice were culled at 16 weeks of age, 6 weeks after transgene induction. Mice were sacrificed by cervical dislocation and decapitation between 12.00–17.00 h for collection of trunk blood. Both femora were excised and fixed in 4% PBS-buffered paraformaldehyde for 16 h at 4°C. Right femora were bisected transversely at the midpoint of the long axis. After dehydration, distal halves were embedded undecalcified in methyl-methacrylate (Medim-Medizinische Diagnostik, Giessen, Germany).

Sagittal 5 µm sections were stained and evaluated as previously [29]. Analysis of cancellous bone volume (BV/TV, %), trabecular thickness (Tb.Th, µm), and number (Tb.N,/mm) was carried out on modified von Kossa stained sections. To assess bone formation indices, s.c. calcein (20 mg/kg) (Sigma Chemical Company, St Louis, USA) was given 10 and 3 days prior to collection. Mineralizing surface (MS, %) and mineral apposition rate (MAR, µm/d) were measured from unstained sections, and bone formation rate was calculated (BFR  =  MS/BS * MAR, µm^2^/µm/d) [29]. Bone resorption indices, osteoclast surface (Oc.S, %) and number (Oc.N,/mm), were estimated in tartrate-resistant acid phosphatase stained sections [30]. All cancellous measurements were conducted in a sample area bordering the epiphyseal growth plate, beginning 0.25 mm proximal to the mineralization zone and extending proximally by 4.2 mm, encompassing all the cancellous bone within the cortices [31]. Cortical mineral apposition rate was measured at the posterior endosteal mid-shaft, extending 1000 µm proximal from the mid-femora.

### Micro-computed Tomography (Micro-CT)

A Skyscan 1174 scanner and associated analysis software (Skyscan, Aartselaar, Belgium) were used to examine 3-dimensional bone structure as previously described [32]. Following fixation, bone was scanned in a plastic tube filled with 70% ethanol. A 0.5 mm aluminum filter was applied to the 50 kV X-ray source, exposure time of 3600 ms and sharpening at 40%. Distal femora were scanned at a 6.2 µm pixel resolution acquired over an angular range of 180°, with a rotation step of 0.4°. Following reconstruction, cortical bone analyses were carried out in 150 slices (0.93 mm) starting 4.5 mm proximal from the distal growth plate using CT-Analyser software (Skyscan). In PYYtg mice, femur length was different between genotypes, thus cortical bone region was adjusted for femur length. For vertebrae, slices between the rostral and caudal growth-plates were selected, with 10 slices offset from the rostral growth-plate and 25 slices offset from the caudal growth-plate. The vertebrae body (L3) was then subdivided into cancellous and cortical components by manual tracing.

### Isolation of Calvarial Osteoblastic Cells

Primary calvarial osteoblasts were isolated as previously described [33]. Briefly, five calvaria from six-day-old mice were then digested using 0.1% Collagenase and 0.2% Dispase II α-MEM without FCS and were shaken for 10 min at 37°C. After incubation the liquid phase, was discarded. This was repeated, adding 1 ml of digestion medium, but the liquid (fraction 2) was collected. This was repeated 4 times. The cells from fractions 2 to 5 were pooled by centrifugation (5 min, 500 g). The pellet was re-suspended in 1 ml α-MEM. The cells were seeded and cultured in 6-well plates. 1.5 ml of α-MEM culture medium containing 10% FCS and antibiotics and 1 ml of cell suspension was added and grown at 37°C in 5% CO2 in air. The medium was replaced every 3 days. The medium was pre-warmed to 37°C before changing. The cells were grown until sub-confluent, trypsinized and plated in 24-well plates.

### Stimulation, Protein Extraction and Western blotting

Primary calvarial cells were serum starved overnight (0.5% FBS) and treated with 50 nM PYY with or without 1 µM of the Y1 receptor antagonist 1229U91 [34] (Sigma-Aldrich) for the indicated times. Protein extracts were prepared as previously described [35]. Proteins were electrophoresed on a 10% SDS polyacrylamide gels, transferred to nitrocellulose membrane and probed with p44/42 MAPK (Erk1/2) antibody (Cell Signaling Technology). Anti-mouse/rabbit IgG HRP conjugate (Promega) was used as a secondary antibody. Bands were detected by ECL (GE Healthcare Australia). Densitometric analysis of western blots were performed using ImageJ software (http://rsbweb.nih.gov/ij/) quantifying mean gray values subtracted from background.

### Total RNA Extraction and RT-PCR of Bone Marrow Monocytes (BMM) and RANKL-Induced Osteoclasts

Age and sex matched WT (C57BL/6) mice were sacrificed and hindlimbs were dissected. The bone marrow was flushed from the tibia and femur with a 21G needle syringe in α-MEM and the cells were resuspended in fresh complete α-MEM (10% FCS, Pen-Strep, GlutaMax) with 10 ng/ml human M-CSF (R&D systems) for BMM culture for three days in T-75 flasks. Non-adherent cells were discarded through media changes. Adherent cells were released by trypsin and seeded at a density of 5×10^4^ cells per well in 6-well plates. A RANKL time course was performed at the allocated time points (0, 2, 3, 4, 5 days) with 50 ng/ml murine sRANKL (Peprotech). Total RNA was isolated using Trizol Reagent (Invitrogen) according to manufacturer instructions and Reverse Transcription was performed using the Superscript III First-Strand Synthesis System (Invitrogen). RT-PCR was performed with the cyclic conditions: 94°C, 40 sec; 55°C, 40 sec; 72°C, 45 sec for 30 cycles with the following primers: GAPDH, Forward: ACTTTGTCAAGCTCATTTCC, Reverse: TGCAGCGAACTTTATTGATG; Y1R, Forward: CTCGCTGGTTCTCATCGCTGTGGAACGG, Reverse: GCGAATGTATATCTTGAAGTAG; Y2R, Forward: TCCTGGATTCCTCATCTGAG, Reverse: GGTCCAGAGCAATGACTGTC; Y4R, Forward: TCTACAGACAGTAGACCAGG, Reverse: GTAGGTTGGTCACATTGGAC; Y5R, Forward: GGGCTCTATACATTTGTAAGTCTTCTGGG, Reverse: CATGGCTTTGCCGAACATCCACTGATCC; y6R, Forward: GGAGGGATGGTTATTGTGAC, Reverse: GTTGTTGCTCTTGCCACTGG; Cathepsin K, Forward: GGGAGAAAAACCTGAAGC, Reverse: ATTCTGGGGACTCAGAGC; Calcitonin Receptor, Forward: TGGTTGAGGTTGTGCCCA, Reverse: CTCGTGGGTTTGCCTCATC. PCR products were analyzed by agarose gel electrophoresis.

### MTS Cell Proliferation Assay on Bone Marrow Stromal Cells (BMSCs)

Age and sex matched WT and germline Y1R^−/−^ mice were sacrificed and hindlimbs were dissected for bone marrow extraction. The bone marrow was flushed from the tibia and femur with a 21G needle syringe in α-MEM and the cells were resuspended in fresh complete α-MEM (10% FCS, Pen-Strep, GlutaMax) and cultured for seven days in T-75 flasks or 10 cm dishes. Media was changed every two days to remove non-adherent cells. After seven days of culture, the cells were trypsinized and plated at 8×10^3^ cells per well in 96-well plates to be cultured for two days before differentiation in osteogenic media (50 µg/ml Ascrobic acid, 10 mM β-glycerophosphate) with 25–100 nM concentrations of hPYY 1–36aa (Sigma) for 5 days. The Promega CellTiter 96® AQueous MTS cell proliferation assay (Cat# G5421) was used according to manufacturer protocol (Promega). Briefly, osteogenic media was replaced with complete α-MEM before 20 µl of MTS-PMS reagent was added per well (100 µl of media) and incubated for four hours at 37°C. Cell free wells were used as blanks. Absorbance at 490 nm was quantified by spectrophotometer.

### Statistical Analysis

Data are expressed as means ± standard error (SEM). Differences between genotypes were assessed by two-tailed students T-test using GraphPad Prism 5 (Version 5.0a, GraphPad Software, Inc). For all statistical analyses, *p* values below 0.05 were considered statistically significant.

## Results

### Lack of PYY Leads to Greater Bone Formation in Mice

**Figure 1:**
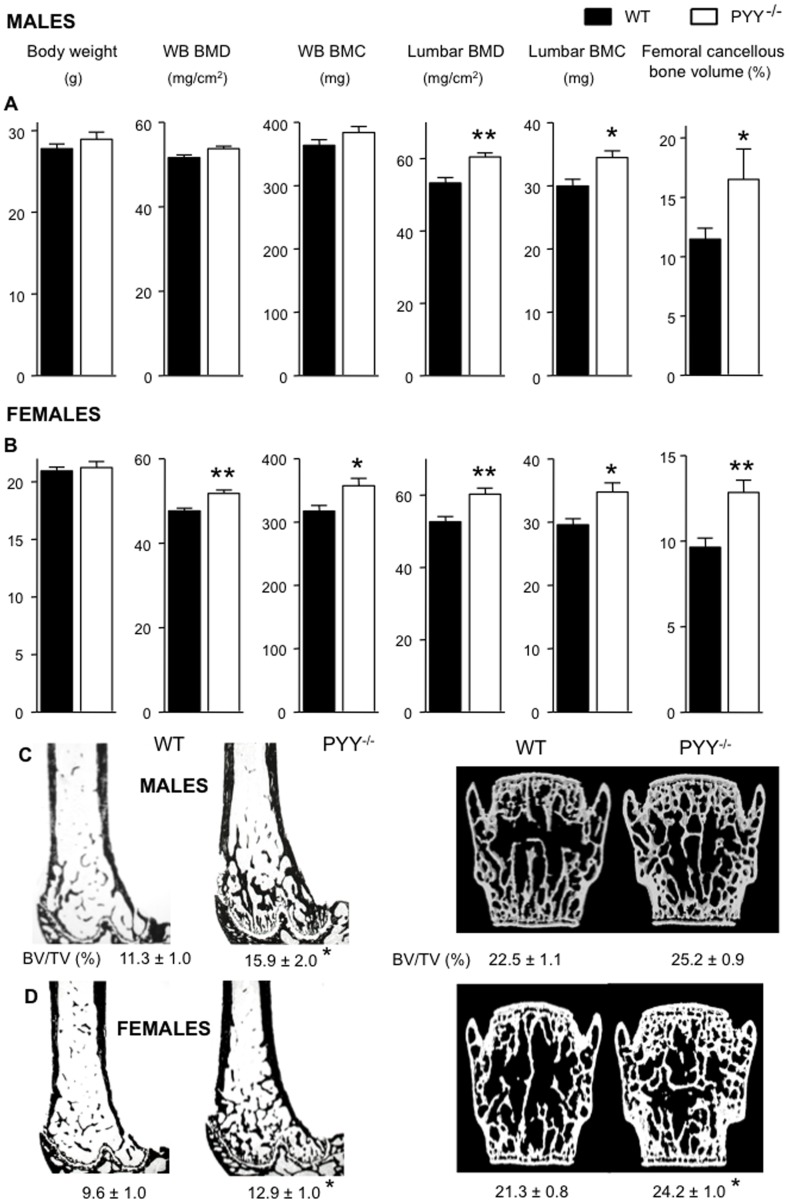
Greater bone mass in PYY^−/−^ mice. Male (A) and female (B) PYY^−/−^ mice display no difference in body weight, but greater whole body BMD, whole body BMC in female mice. Regional analysis revealed greater lumbar BMD and BMC in PYY^−/−^ of both genders. Representative images of distal femur histological sections and 3D surface rendered models of µCT scanned lumbar vertebra of male (C) and female (D) mice showing greater cancellous bone volume in the distal femoral metaphysis of PYY^−/−^ mice in both genders and in the lumbar vertebral body of female PYY^−/−^ mice (D). Mean ± SEM of 7–18 mice per group are shown. *p<0.05, **88p<0.005 versus wild-type (WT).

In order to investigate the effect of PYY deficiency on bone in the absence of any confounding effects on body weight, knockout mice at the age of 14 weeks were studied, prior to the onset of greater body weight [26]. Dual energy X-ray absorptiometry (DXA) revealed greater whole body BMD and BMC in PYY^−/−^ mice compared to wild-type controls, significantly so in females (Figure 1A, B). In the lumbar vertebrae, PYY^−/−^ mice of both genders exhibited significantly greater BMD and BMC than wild-type, without significant differences in femur length and lumbar vertebral height between genotypes (data not shown). Histomorphometric analysis revealed significantly greater cancellous bone volume in PYY^−/−^ mice of both genders at the distal femoral metaphysis, as evident in tissue sections stained for mineralized tissue (Figure 1C, D). In female mice, PYY deletion also increased cancellous bone volume in the lumbar vertebral body as measured by µCT (Figure 1D).

**Figure 2:**
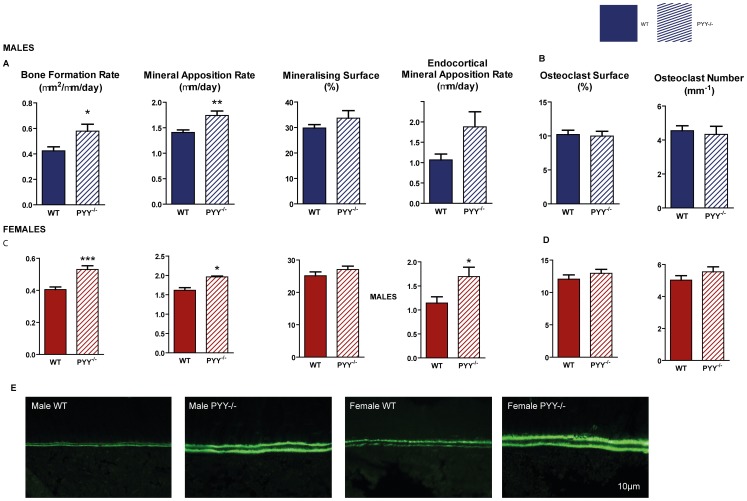
Bone cell activities in the femora of PYY^−/−^ mice. In the distal femoral metaphysis, lack of PYY signaling results in greater bone formation rate (A,C) due to greater mineral apposition rate (MAR) with no change in mineralizing surface (C,I) in both genders. Endocortical MAR at the mid femur was also greater in PYY^−/−^ compared to wild-type. Parameters of cancellous bone resorption as indicated by osteoclast surface and number (B,D) are similar between PYY^−/−^ and wild-type controls. Representative images of endocortical MAR are shown in E. Scale bar represents 10 µm. Mean ± SEM of 5–18 mice per group are shown. *p<0.05, **p<0.005, ***p<0.001 versus wild-type (WT).

The increased cancellous bone volume in the distal femoral metaphysis was associated with enhanced bone formation in male and female PYY^−/−^ compared to wild-type (Figure 2 A, C). This was via greater speed (mineral apposition rate, MAR) but not extent (mineralizing surface, MS) of bone formation. Similarly, endocortical MAR at the mid-femur was greater in PYY^−/−^ (representative images Figure 2E), although cortical structure was not different between genotypes (Table S2). No change was observed in osteoclast activity, as indicated by osteoclast surface (Figure 2B, D) or osteoclast number in the distal femoral metaphysis of PYY^−/−^ versus wild type mice of either gender.

### Adult Onset PYY Over-expression Reduces Bone Formation and Increases Bone Resorption

**Figure 3:**
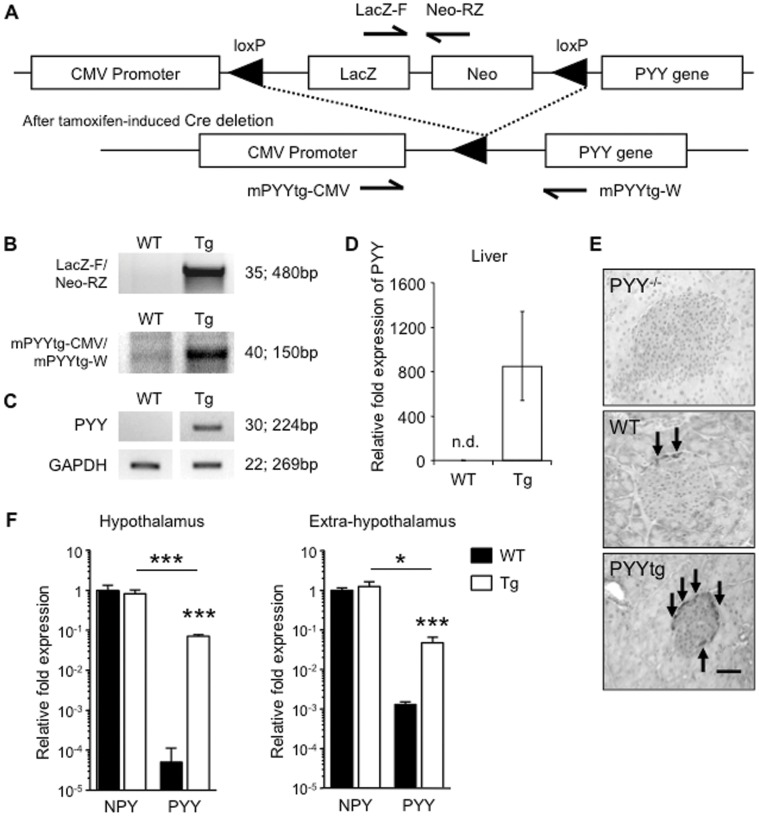
Evaluation of PYY overproduction. As shown in the schematic diagram of the PYY transgene construct (A), liver genomic DNA was analyzed for presence of PYY transgene construct (B) and deletion of stop cassette (hence activation of PYY transgene) by southern analysis. Hepatic PYY mRNA expression was detected only in PYYtg mice and not littermate controls as shown by RT?PCR (C) and qPCR (D). PYY staining (brown) is more intense in the islets of Langerhans in the pancreas of PYY transgenic than wild-type littermates (E). PYY mRNA levels are greater in PYYtg hypothalamus and the rest of the brain, compared to wild-type littermates, but are lower compared to NPY mRNA levels of the same genotype (F). Number of PCR cycles is indicated at right, followed by size of PCR product (B-C). Values shown are normalised to ribosomal protein L13 (RPL-13) levels and relative to wild-type expression (D, F). n.d., not detected (E). Scale bar represents 50 µm (E). Pictures are representative of staining observed in tissues obtained from 16-week-old mice (2?3 mice per group) (E). *p<0.05, ***p<0.001 versus wild-type (WT).

Having established that lack of PYY increased bone formation, we next investigated whether increased levels of PYY, as commonly seen under anorexic conditions [11], [14], have opposing effects. Elevated PYY is lethal during embryonic development [36], thus PYY over-expression was induced in adult animals via a Cre-recombinase activated mechanism (Figure 3A). For this purpose, inducible PYY transgenic mice (PYYtg^lox/lox^) were crossed with mice expressing Cre-recombinase under a tamoxifen-inducible promoter located within the ROSA26 locus (ROSACre). Transgene expression in PYY^lox/creERT2^ mice (from now on called PYYtg) was induced in 10 week-old mice, with tissues collected at 16 weeks of age. Tamoxifen injected PYYtg^lox/lox^ (from now on called WT) mice were used as controls. The presence and induction of the PYY transgene were confirmed by PCR on genomic DNA (Figure 3B). PYY mRNA over-expression was identified in the liver of PYYtg, but not in the liver from wild-type mice (Figure 3C, D). Immunohistochemical staining revealed increased staining for PYY protein in the islets of Langerhans in the pancreas of PYYtg mice compared to wild-type or PYY^−/−^ (Figure 3E). PYY mRNA was also over-expressed in the hypothalamus and, to a lesser extent, in the rest of the brain (extra-hypothalamus) (Figure 3F). However, central PYY mRNA levels were still 10-fold lower compared to central mRNA levels of NPY, the main ligand for Y receptors in the brain, identifying NPY as the major hypothalamic signal in PYYtg mice, and suggesting that PYY over-expression in this transgenic model impacts predominantly peripheral Y receptor signaling also demonstrated in a recent report showing altered glucose metabolism in this model [37].

**Figure 4:**
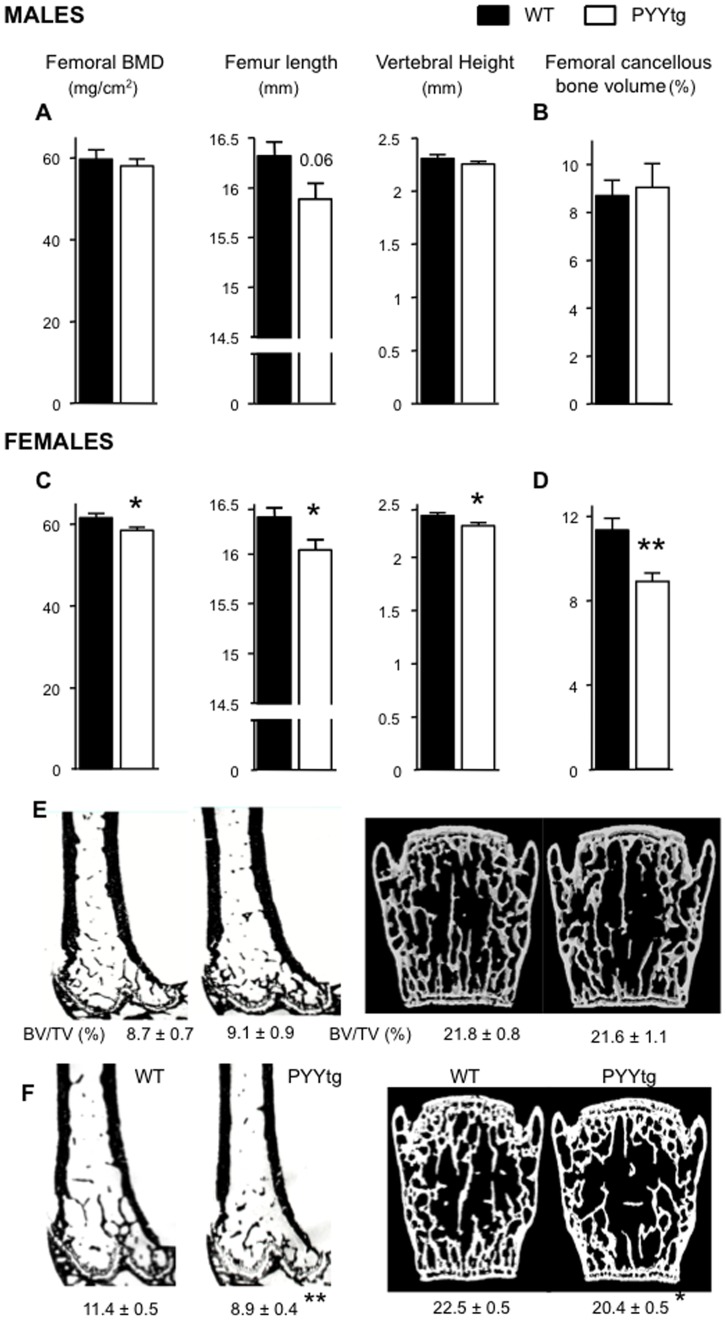
Lower bone mass and reduced bone size in female PYYtg mice. Although male PYYtg mice are similar to wild-type littermates (A-B), female PYYtg mice have lower femoral BMD, femur length, vertebral height and cancellous bone volume of distal femoral metaphysis (C-D); representative images from male (E) and female (F) mice of distal femoral histological sections and 3D model images of µCT scanned lumbar vertebra. Mean ± SEM of 4–12 mice per group are shown. *p<0.05, **p<0.05 versus wild-type (WT).

Consistent with our hypothesis that PYY inhibits bone mass, PYYtg mice displayed a skeletal phenotype essentially opposing PYY^−/−^. Lean mass was lower in female PYYtg littermates prior to tamoxifen (Table S3), the change in body composition was similar between PYYtg and WT 6 weeks post-injection (Table S4). At cull, isolated femoral BMD, femoral length and vertebral height were significantly lower in female PYYtg than control, with similar trends in male PYYtg (Figure 4A, C). Furthermore, a reduction in cancellous bone volume was observed specifically in female but not male PYYtg at the distal femora as well as lumbar vertebrae (Figure 4B, D, E, F).

**Figure 5:**
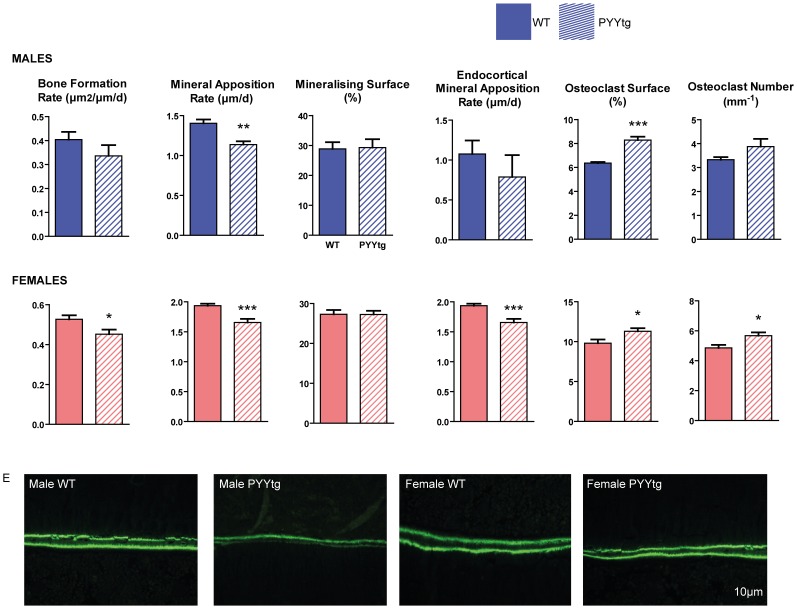
Bone cell activities in the femora of PYYtg mice. Although cancellous bone volume is lower in female but not male PYYtg, alterations in bone cell activities are observed in both genders. Mineral apposition rate (MAR) was reduced in both genders (A,C), with bone formation rate reduced only in females, with no change in mineralizing surface. Endocortical MAR at the mid femur was reduced in female PYYtg compared to wild-type. Osteoclast surface was greater in both genders of PYYtg and osteoclast number only greater in females. Representative images of endocortical MAR are shown in E. Scale bar represents 10 µm. Mean ± SEM of 4–12 mice per group are shown. *p<0.05, **p<0.005, ***p<0.001 versus wild-type (WT).

Analysis of the distal femoral metaphysis of PYYtg revealed changes in both bone resorption and formation, indicating a comprehensive regulation of the bone remodeling cycle by PYY. Osteoblast activity (MAR) was significantly reduced in both male and female PYYtg mice compared to wildtype. This is consistent with the opposite phenotype of enhanced MAR observed in PYY^−/−^ mice, and indicates a negative association between PYY and osteoblast activity. Furthermore, cancellous bone formation rate and mid-femoral endocortical MAR were significantly lower in female but not male PYYtg (Figure 5A, C, E), suggesting a more pronounced effect of PYY on bone, in female mice. In cortical bone, alterations in cortical MAR in female PYYtg versus control mice were associated with structural changes as indicated by reductions in total cross-sectional area, cortical bone area and mean polar moment of inertia, an index of bone strength (Table S5). In males, mid-femoral cortical changes were consistent with females; however they did not reach significance.

In addition to modulating osteoblast activity, PYY over-expression also affected bone resorbing osteoclasts. PYYtg mice displayed greater osteoclast surface measured in the femoral metaphysis of both genders (Figure 5B, D) and greater osteoclast number in females only mice, despite comparable elevations of osteoclast number in male PYYtg. Interestingly, these effects of elevated PYY expression on bone resorption were not observed in PYY^−/−^ mice.

**Figure 6:**
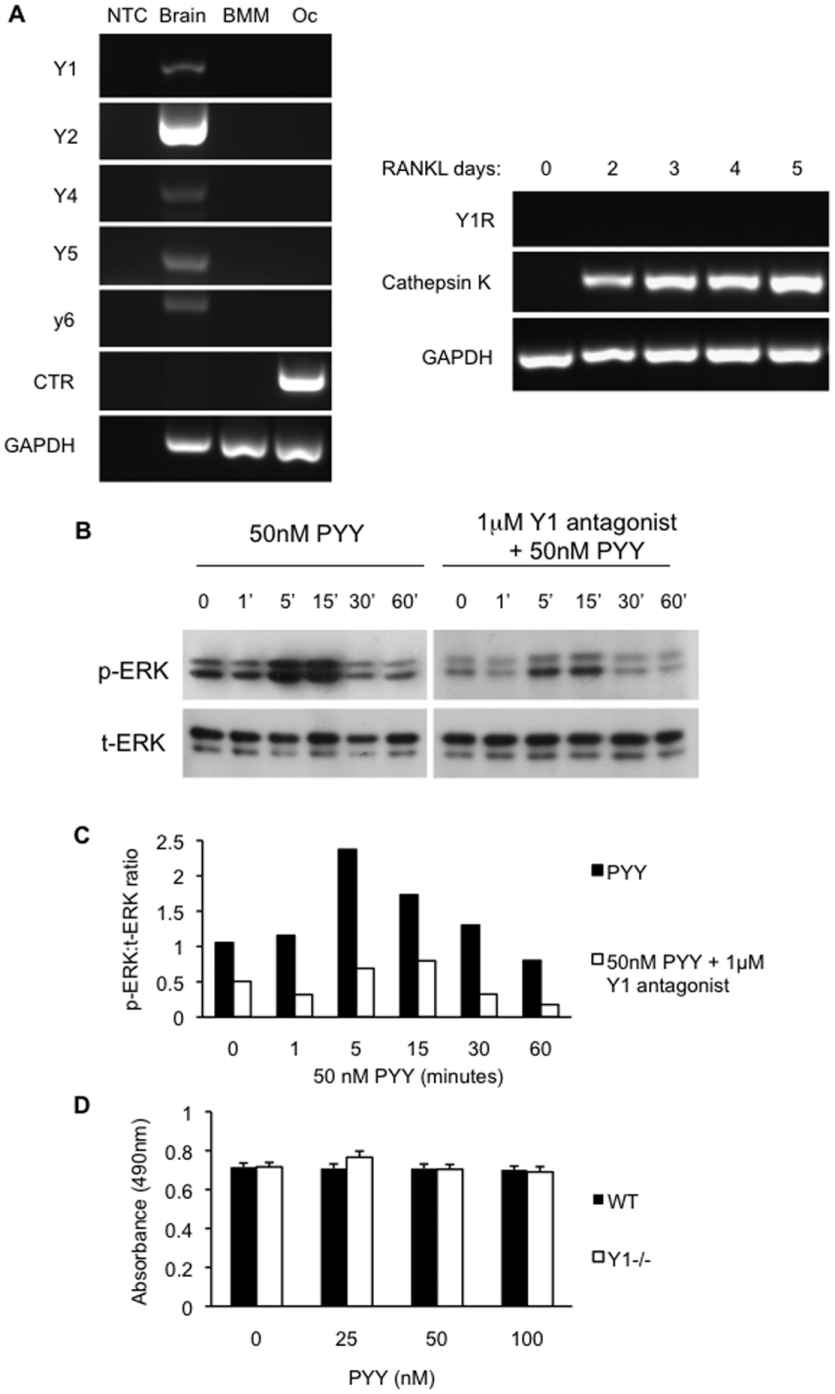
PYY signalling in osteoblasts via Y1 receptors. Y receptor gene expression was evident in brain tissue, but was absent in bone marrow macrophages (BMM) or cultured osteoclasts (Oc) from wild type mice(A). Y1R was not detected at any time point throughout RANKL-induced osteoclast differentiation (A). Calcitonin Receptor (CTR) and Cathepsin K gene expression were used as osteoclast markers. NTC, no template control. PYY induced signaling in primary calvarial osteoblasts through strong phosphorylation of ERK, a response which was blunted in the presence of Y1 receptor specific antagonist (1229U91). (B) p-ERK, phosphorylated ERK; t-ERK, total ERK from western blots of calvarial cultures. (C) Western blots were quantified by densitometry of p-ERK and t-ERK bands. Proliferation of osteoblastic cultures from wild type and Y1 receptor null mice were not altered by PYY (D). Mean ± SEM of 4 replicates per group are shown.

The alteration in osteoclast indices suggested the possibility of a direct action of PYY on these cells. To examine this, Y receptor expression was assessed in osteoclastic cultures from wild type and PYY Tg mice. RT-PCR was performed on mRNA isolated from mature (5 day) cultured osteoclasts employing sets of primers specific for all known Y-receptors (Y1, Y2, Y4, Y5 and y6). Positive signals for all Y-receptors were obtained from mRNA derived from the brain (Figure 6A). In contrast, no signal for any of the receptors was detected in osteoclastic mRNA or from bone marrow macrophages (BMM). Since the Y1R is known to be expressed in myeloid lineage cells [38] a time course study was undertaken to examine the potential for early PYY effects. Y1R expression was absent at all time points through osteoclast maturation confirming that PYY action on bone is purely mediated via signalling on osteoblasts.

### PYY Signals Directly in Osteoblastic Cells via the Y1 Receptor

In order to examine the mechanism by which PYY may regulate bone homeostasis, primary calvarial osteoblast cultures were treated with 50 nM PYY and cellular activation assessed by examination of ERK phosphorylation. Consistent with a direct action of PYY on osteoblasts, ERK phosphorylation was rapidly induced following exposure to PYY (Figure 6B, C). This response was markedly diminished in the presence of the Y1 receptor-specific antagonist (1229U91) [34], the only Y receptor known to be expressed on osteoblasts [39], [40], [41]. This result confirms a direct action of PYY on bone cells through Y1 receptors signalling.

To assess the effect of PYY on osteoblast proliferation primary bone marrow stromal cell (BMSC) cultures were grown in osteogenic media and treated with 50 nM PYY for 5 days in which cell number was assessed by the MTS cell proliferation assay. PYY had no effect upon cell number in either wild type or Y1 receptor null BMSC cultures (Figure 6D). This result is consistent with PYY effects upon osteoblast activity (MAR) not surface extent (MS) evident in both PYY^−/−^ and PYYtg mice.

## Discussion

Recently, our understanding of the interactions between the gut and bone has increased markedly, with the gastrointestinal/skeletal interface emerging as an important regulator of bone mass. In addition to classic pathways involving calcium balance, the gut is now recognized for a far more complex regulatory role in skeletal homeostasis, with a number of gastro-intestinal peptides identified as regulating bone mass [2]. In the present study we demonstrate the critical role for the gut-derived satiety hormone PYY in the maintenance of bone homeostasis. Overproduction of PYY in adult animals reduces bone mass whereas lack of PYY in mice increases bone mass. Importantly, PYY’s role in the maintenance of bone mass involves both arms of the bone remodeling cycle; reducing the formation of bone and increasing bone resorption, displaying the complexity of the osteo-regulatory signals coming from the gut.

Our data demonstrate a suppressive effect of PYY on the activity of bone forming osteoblasts, reducing the rate at which mineralized tissue is formed by these cells. This effect is evident throughout the skeleton, with changes in both the dense, structural cortical bone as well as in the sponge-like and more metabolically active cancellous bone. However, PYY does not appear to alter the production of osteoblastic cells *in vivo*, as indicated by no change in their surface extent. *In vitro* studies indicate that PYY does not affect osteoblast proliferation, a finding consistent with previous studies of Y1R, Y2R and NPY knockout osteoblasts in culture, all of which showed no changes prior to 15 days in culture [30], [40], [41]. In addition to effects on bone formation, PYY altered the surface extent of the bone resorbing osteoclasts. In a cellular change evident only following PYY over expression as osteoclasts were more abundant on the bone surface of PYYtg mice. Interestingly, the lack of Y receptors in osteoclasts suggests that PYY may indirectly affect osteoclast activity. Thus elevation in PYY levels results in dual negative impacts upon the maintenance of bone mass. In keeping with these changes in bone cell activity, loss of PYY signaling was associated with significantly increased bone mass in PYY^−/−^ mice of both genders. Interestingly, in PYYtg mice, bone mass was only reduced in female mice. Cellular changes were consistent across both genders; however, the lack of change in bone mass in males suggests a potential sex-specific influence. Taken together, these findings demonstrate that PYY inhibits osteoblast activity and stimulates osteoclast production in both cancellous and cortical bone, and the skeletal effects of PYY are more prominent in female than in male mice.

These findings provide a possible mechanism for the clinical observation of bone loss in situations associated with elevated circulating PYY. PYY is considered as a potential contributor to the low bone mass of anorexia nervosa, where PYY levels are reported to be increased [14]. Several techniques of bariatric surgery (gastric sleeve and Roux en Y bypass) may also produce a marked elevation in PYY responses [19], [20], and with it a potential for exaggerated bone loss in addition to that associated with weight reduction. Perhaps more importantly, PYY levels have a significant, negative association with bone mass in healthy premenopausal women [13]. This suggests that PYY may play a tonic inhibitory role in bone homeostasis in normal populations, as well as in those with overt changes in energy homeostasis.

In contrast to our present findings, however, a low bone mass phenotype was reported in an independently generated PYY^−/−^ mouse model [6]. Differences between germline knockout models are not unusual and the reason for the discrepant results in these two models may relate to a different design of the targeting vector (insertion of a large reporter gene LacZ versus absence of additional sequences in our model), some variation in the genetic background (the origin/background of the ES cells is not given), and the age at which the analysis of bone was performed (i.e. 3–4 months in our model compared to 6–9 months in the other model). It is also important to note that the PYY knockout model generated by *Wortley* et al (6) has also a very different phenotype in respect to body weight and metabolic parameters compared to our model as well as another one published by Batterham et al [16] both of which showed increased BW and fat mass and altered glucose homeostasis. Together, this could have significant influences on bone mass. Furthermore, although cancellous bone was analyzed by histomorphometry in the vertebrae in the initial study, no bone cell activities or long bone parameters were examined in the previous report (6).

Importantly, our data showing increased bone mass in the absence of PYY and decreased bone mass when PYY is elevated are also consistent with findings from knockout models of Y1 and Y2 receptors [31], [32], [41], [42]. These models have clearly established that bone mass is under the control of the NPY system, indirectly via hypothalamic Y2 receptors [31] and NPY-ergic neurons [28], as well as directly via osteoblastic Y1 receptors [39], [41]. While PYY is ideally placed to signal via both the hypothalamic Y2 receptors and osteoblastic Y1 receptors, *in vitro,* PYY signaling was clearly evident in osteoblasts, stimulating the MAPK pathway, as has been demonstrated for NPY [39]. Importantly, this effect was markedly blunted by Y1 receptor antagonism, indicating direct PYY signaling in osteoblasts via the Y1 receptor. Based upon previous models, activation of either Y1 or Y2 receptors will lead to inhibition of bone formation, consistent with reduced osteoblast activity seen following PYY overproduction. Importantly, PYY is processed via dipeptidyl peptidase-4 (DPP IV) to the shorter version, PYY_3–36_, which shows an altered pharmacological profile. PYY_3–36_, like the full length PYY, is able to signal through Y2 receptors, but loses its affinity for the Y1 receptor. This may ensure that the satiety function of this gut peptide is preserved and prolonged, while peripheral Y1-mediated actions, such as the inhibition of bone formation, are attenuated. The ratio of the full-length versus the truncated version of PYY is likely to be critical in the normal control of bone mass. From a clinical perspective, inhibiting the action of DPP IV to prevent inactivation of another beneficial gut peptide, glucagon-like peptide 1 (GLP-1), might have negative side effects on bone mass due to concomitant stabilization of the Y1-active full length PYY.

In summary, detailed analyses using both PYY knockout and PYY transgenic mouse models demonstrate that PYY exerts a negative influence upon the maintenance of bone mass, and mediates its skeletal effects via modulation of osteoblast and osteoclast activity. Importantly, these findings suggest that the changes in circulating PYY levels observed in clinical conditions, such as anorexia nervosa, amenorrhea and patients who have undergone bariatric surgery [2], may contribute to the alterations in bone mass in these patients. Moreover, gut derived PYY may represent a significant regulatory factor in the maintenance of bone mass in normal populations [13], reinforcing the importance of the gut-derived endocrine system in skeletal homeostasis. Modulation of PYY may therefore offer novel avenues for control of bone mass in specific patient groups, as well as providing insight into the role of the gut in wider homeostatic processes.

## Supporting Information

Table S1 **PCR used for genotyping and expression studies in PYY transgenic mice.** Nucleotide sequence, PCR conditions and band size for respective genes for genotyping and expression quantification.
(DOC)

Table S2 **Cortical bone phenotype in the mid femora of male and female PYY−/− mice.** Means ± SE of 4–18 mice per group shown. a indicates p<0.05, b indicates p<0.10 versus wild-type.
(DOC)

Table S3 **Baseline characteristic (before tamoxifen injection at 8 weeks of age) of PYYtgROSACre and wild-type littermates.** Means ± SE of 5–9 mice per group. a indicates p<0.05 versus wild-type.
(DOC)

Table S4 **Similar change in body composition of PYYtgROSACre and wildtype littermates before and after tamoxifen injection as measured by dual X-ray absorptiometry.** Means ± SE of 5–9 mice per group.
(DOC)

Table S5 **Cortical bone phenotype in the mid femora of male and female PYYtgROSACre mice.** Means ± SE of 4–12 mice per group shown. a indicates p<0.05, b indicates p<0.10 versus wild-type.
(DOC)
