# Supplementary material for: Peptide YY Regulates Bone Remodeling in Mice: A Link between Gut and Skeletal Biology
Source: PLoS One. 2012 Jul 6;7(7):e40038. doi: 10.1371/journal.pone.0040038 (PMC3391226; doi:10.1371/journal.pone.0040038)
Supplement: Table S1 — PCR used for genotyping and expression studies in PYY transgenic mice. Nucleotide sequence, PCR conditions and band size for respective genes for genotyping and expression quantification. (DOC) [file pone.0040038.s001.doc]

Table S1. PCR used for genotyping and expression studies in PYY transgenic mice.

| **Primer** | **Nucleotide sequence (5’ 🡪 3’)** | **PCR condition** | **Band (bp)** |
| --- | --- | --- | --- |
| **Genotyping** |  |  |  |
| LacZ-F | GGTAAACTGGCTCGGATTAG | 94°C - 1min, 60°C - 1min, 72°C - 45sec | 480 |
| Neo-RZ | TCATAGCCGAATAGCCTCTC |
| mPYYtg-CMV | CGTGCTGGTTATTGTGCTGTC | 94°C - 1min, 56°C - 1min, 72°C - 45sec | 150 |
| mPYY-W | TCTAGACATCTAGGAGGAAAGCTTTG |
| **RT-PCR** |  |  |  |
| PYY-sense | ACGGTCGCAATGCTGCTAAT | 94°C - 40sec, 65°C - 40sec, 72°C - 45sec | 224 |
| PYY-antisense | AAACCTTCTGGCCTGAAGGG |
| GAPDH-sense | ACTTTGTCAAGCTCATTTCC | 94°C - 40sec, 57°C - 40sec, 72°C - 45sec | 269 |
| GAPDH-antisense | TGCAGCGAACTTTATTGATG |
| **qPCR** |  |  |  |
| PYY-sense | TTCACAGACGACAGCGACA |  |  |
| PYY-antisense | CACCACTGGTCCAAACCTTC |  |  |
| ribosomal protein L19-sense | CTCGTTGCCGGAAAAACA |  |  |
| ribosomal protein L19-antisense | TCATCCAGGTCACCTTCTCA |  |  |

#### Nucleotide sequence, PCR conditions and band size for respective genes for genotyping and expression quantification.
