# Supplementary material for: Peptide YY Regulates Bone Remodeling in Mice: A Link between Gut and Skeletal Biology
Source: PLoS One. 2012 Jul 6;7(7):e40038. doi: 10.1371/journal.pone.0040038 (PMC3391226; doi:10.1371/journal.pone.0040038)
Supplement: Table S2 — Cortical bone phenotype in the mid femora of male and female PYY−/− mice. Means ± SE of 4–18 mice per group shown. a indicates p<0.05, b indicates p<0.10 versus wild-type. (DOC) [file pone.0040038.s002.doc]

Table S2. Cortical bone phenotype in the mid femora of male and female PYY^-/-^ mice.

| **MALES** | **WT** | **PYY^‑/‑^** |
| --- | --- | --- |
| Total cross-sectional area (mm^2^) | 1.86 ± 0.07 | 1.73 ± 0.06 |
| Cortical bone area (mm^2^) | 0.92 ± 0.03 | 0.90 ± 0.04 |
| Marrow area (mm^2^) | 0.94 ± 0.04 | 0.83 ± 0.03 |
| Cortical area fraction (%) | 49.8 ± 0.9 | 52.1 ± 0.7 |
| Cortical thickness (mm) | 226 ± 3 | 235 ± 7 |
| Periosteal perimeter (mm) | 5.29 ± 0.10 | 5.04 ± 0.10 |
| Endosteal perimeter (mm) | 4.00 ± 0.15 | 3.61 ± 0.04 |
| Mean polar moment of inertia (mm^4^) | 0.44 ± 0.03 | 0.38 ± 0.03 |
| **FEMALES** | **WT** | **PYY^‑/‑^** |
| Total cross-sectional area (mm^2^) | 1.79 ± 0.03 | 1.68 ± 0.04 |
| Cortical bone area (mm^2^) | 0.87 ± 0.02 | 0.84 ± 0.01 |
| Marrow area (mm^3^) | 0.92 ± 0.03 | 0.84 ± 0.03 |
| Cortical area fraction (%) | 48.7 ± 1.2 | 50.1 ± 0.9 |
| Cortical thickness (μm) | 218 ± 5 | 219 ± 4 |
| Periosteal perimeter (mm) | 5.15 ± 0.04 | 4.97 ± 0.07 ^b^ |
| Endosteal perimeter (mm) | 3.76 ± 0.05 | 3.56 ± 0.08 ^b^ |
| Mean polar moment of inertia (mm^4^) | 0.39 ± 0.01 | 0.35 ± 0.02 |

#### Means ± SE of 4‑18 mice per group shown. *^a^* indicates *p* < 0.05, *^b^* indicates *p* < 0.10 versus wild-type**.**

#### 
