# Supplementary material for: Peptide YY Regulates Bone Remodeling in Mice: A Link between Gut and Skeletal Biology
Source: PLoS One. 2012 Jul 6;7(7):e40038. doi: 10.1371/journal.pone.0040038 (PMC3391226; doi:10.1371/journal.pone.0040038)
Supplement: Table S3 — Baseline characteristic (before tamoxifen injection at 8 weeks of age) of PYYtgROSACre and wild-type littermates. Means ± SE of 5–9 mice per group. a indicates p<0.05 versus wild-type. (DOC) [file pone.0040038.s003.doc]

#### Table S3. Baseline characteristic (before tamoxifen injection at 8 weeks of age) of PYYtgROSACre and wild-type littermates.

| **MALES** | **PYYtgROSA WT** | **PYYtgROSACre** |
| --- | --- | --- |
| Body weight (g) | 22.2 ± 0.5 | 21.2 ± 0.5 |
| Fat mass (g) | 2.9 ± 0.1 | 2.9 ± 0.0 |
| Lean mass (g) | 17.9 ± 0.5 | 17.0 ± 0.5 |
| Whole body BMD (mg/cm^2^) | 47.2 ± 0.7 | 46.0 ± 0.6 |
| Whole body BMC (mg) | 297 ± 11 | 286 ± 8 |
| **FEMALES** | **PYYtgROSAWT** | **PYYtgROSACre** |
| Body weight (g) | 17.8 ± 0.3 | 17.1 ± 0.4 |
| Fat mass (g) | 2.7 ± 0.1 | 2.9 ± 0.1 |
| Lean mass (g) | 13.8 ± 0.2 | 12.8 ± 0.3 ^a^ |
| Whole body BMD (mg/cm^2^) | 40.8 ± 0.5 | 40.7 ± 1.1 |
| Whole body BMC (mg) | 238 ± 6 | 234 ± 8 |

#### Means ± SE of 5‑9 mice per group. *^a^* indicates *p* < 0.05 versus wild-type**.**

#### 
