# Supplementary material for: Peptide YY Regulates Bone Remodeling in Mice: A Link between Gut and Skeletal Biology
Source: PLoS One. 2012 Jul 6;7(7):e40038. doi: 10.1371/journal.pone.0040038 (PMC3391226; doi:10.1371/journal.pone.0040038)
Supplement: Table S4 — Similar change in body composition of PYYtgROSACre and wildtype littermates before and after tamoxifen injection as measured by dual X-ray absorptiometry. Means ± SE of 5–9 mice per group. (DOC) [file pone.0040038.s004.doc]

Table S4. Similar change in body composition of PYYtgROSACre and wildtype littermates before and after tamoxifen injection as measured by dual X-ray absorptiometry.

| Compared to before induction | | Male | |  | Female | |
| --- | --- | --- | --- | --- | --- | --- |
|  |  | PYYtgROSAWT | PYYtgROSACre |  | PYYtgROSAWT | PYYtgROSACre |
| Change in body weight | (g) | 5.7 ± 0.4 | 5.8 ± 0.5 |  | 4.5 ± 0.3 | 4.2 ± 0.2 |
|  | (% BW) | 25 ± 2 | 27 ± 3 |  | 25 ± 2 | 24 ± 2 |
| Change in fat mass | (g) | 1.2 ± 0.2 | 1.2 ± 0.3 |  | 0.8 ± 0.2 | 0.8 ± 0.2 |
|  | (% BW) | 5.2 ± 0.9 | 5.6 ± 1.0 |  | 4.4 ± 0.9 | 4.5 ± 1.4 |
| Change in lean mass | (g) | 3.9 ± 0.5 | 4.2 ± 0.6 |  | 3.3 ± 0.2 | 3.2 ± 0.2 |
|  | (% BW) | 17 ± 2 | 19 ± 3 |  | 18 ± 1 | 19 ± 1 |
| Change in WB BMD | (mg/cm2) | 5.9 ± 0.7 | 5.3 ± 0.6 |  | 11.4 ± 0.6 | 10.3 ± 0.9 |
| (% baseline) | | 12 ± 1 | 11 ± 1 |  | 28 ± 2 | 26 ± 3 |
| Change in WB BMC | (mg) | 91 ± 14 | 63 ± 10 |  | 114 ± 5 | 95 ± 8 |
|  | (% BW) | 0.39 ± 0.06 | 0.29 ± 0.05 |  | 0.64 ± 0.03 | 0.56 ± 0.05 |

#### Means ± SE of 5‑9 mice per group.
