# Supplementary material for: Peptide YY Regulates Bone Remodeling in Mice: A Link between Gut and Skeletal Biology
Source: PLoS One. 2012 Jul 6;7(7):e40038. doi: 10.1371/journal.pone.0040038 (PMC3391226; doi:10.1371/journal.pone.0040038)
Supplement: Table S5 — Cortical bone phenotype in the mid femora of male and female PYYtgROSACre mice. Means ± SE of 4–12 mice per group shown. a indicates p<0.05, b indicates p<0.10 versus wild-type. (DOC) [file pone.0040038.s005.doc]

Table S5. Cortical bone phenotype in the mid femora of male and female PYYtgROSACre mice.

| **MALES** | **PYYtgROSAWT** | **PYYtgROSACre** |
| --- | --- | --- |
| Total cross-sectional area (mm^2^) | 2.51 ± 0.09 | 2.25 ± 0.10 ^b^ |
| Cortical bone area (mm^2^) | 0.98 ± 0.04 | 0.90 ± 0.03 |
| Marrow area (mm^2^) | 1.52 ± 0.06 | 1.36 ± 0.08 |
| Cortical area fraction (%) | 39.3 ± 1.0 | 40.0 ± 1.3 |
| Cortical thickness (mm) | 199 ± 6 | 192 ± 5 |
| Periosteal perimeter (mm) | 6.14 ± 0.11 | 5.83 ± 0.13 ^b^ |
| Endosteal perimeter (mm) | 4.95 ± 0.11 | 4.67 ± 0.15 |
| Mean polar moment of inertia (mm^4^) | 0.67 ± 0.05 | 0.55 ± 0.04 ^b^ |
| **FEMALES** | **PYYtgROSAWT** | **PYYtgROSACre** |
| Total cross-sectional area (mm^2^) | 1.80 ± 0.03 | 1.70 ± 0.03 ^a^ |
| Cortical bone area (mm^2^) | 0.92 ± 0.02 | 0.86 ± 0.01 ^a^ |
| Marrow area (mm^3^) | 0.88 ± 0.02 | 0.84 ± 0.03 |
| Cortical area fraction (%) | 51.3 ± 0.8 | 50.7 ± 0.8 |
| Cortical thickness (μm) | 232 ± 4 | 224 ± 3 |
| Periosteal perimeter (mm) | 5.17 ± 0.05 | 5.06 ± 0.06 |
| Endosteal perimeter (mm) | 3.70 ± 0.05 | 3.62 ± 0.06 |
| Mean polar moment of inertia (mm^4^) | 0.41 ± 0.01 | 0.37 ± 0.01 ^a^ |

#### Means ± SE of 4‑12 mice per group shown. *^a^* indicates *p* < 0.05, *^b^* indicates *p* < 0.10 versus wild-type**.**
